# Preclinical Evaluation of Radium-223 and Immune Checkpoint Inhibitors Using an Immune-Competent Model of Prostate Cancer Bone Metastases

**DOI:** 10.3390/precisoncol1010005

**Published:** 2026-03-02

**Authors:** Cynthia Lilieholm, Adedamola O. Adeniyi, Ohyun Kwon, Jen Zaborek, Caroline P. Kerr, Hansel Comas Rojas, Malick Bio Idrissou, Carolina A. Ferreira, Paul A. Clark, Won Jong Jin, Joseph J. Grudzinski, Amy K. Erbe, Reinier Hernandez, Bryan Bednarz, Zachary S. Morris, Jamey P. Weichert

**Affiliations:** 1 Department of Radiology, University of Wisconsin School of Medicine and Public Health, University of Wisconsin-Madison, Madison, WI 53705, USA; 2 Department of Pharmaceutical Sciences, University of Wisconsin School of Pharmacy, University of Wisconsin-Madison, Madison, WI 53705, USA; 3 Department of Medical Physics, University of Wisconsin School of Medicine and Public Health, University of Wisconsin-Madison, Madison, WI 53705, USA; 4 Department of Biostatistics and Medical Informatics, University of Wisconsin School of Medicine and Public Health, University of Wisconsin-Madison, Madison, WI 53705, USA; 5 Department of Human Oncology, University of Wisconsin School of Medicine and Public Health, University of Wisconsin-Madison, Madison, WI 53705, USA

**Keywords:** radium-223 dichloride, alpha-particle therapy, prostate cancer bone metastasis, radiopharmaceutical therapy, immune checkpoint inhibitors, microdosimetry

## Abstract

**Rationale:**

Radium-223 dichloride (^223^RaCl_2_) is an FDA-approved alpha-emitting radiopharmaceutical that targets bone metastases in metastatic castration-resistant prostate cancer (mCRPC). This study investigates the therapeutic and immunological effects of combining ^223^RaCl_2_ with immune checkpoint inhibitors (ICIs) in a clinically relevant, immunocompetent murine model of prostate cancer bone metastasis.

**Methods:**

Luciferase-expressing MyC-CaP prostate cancer cells were implanted intratibially into FVB mice to establish bone metastases. Mice were treated with escalating doses of ^223^RaCl_2_ (0.04–0.27 μCi) alone or a single dose combined with anti-CTLA-4 and anti-PD-L1 ICIs. Tumor growth was monitored using bioluminescence imaging. Micro-CT, alpha camera imaging, histology, and qPCR were used to assess bone remodeling, radiopharmaceutical distribution, immune infiltration, and gene expression. Ex vivo biodistribution and blood analyses quantified tissue uptake and toxicity.

**Results:**

Escalating doses of ^223^RaCl_2_ did not significantly inhibit tumor growth or improve survival. Biodistribution and imaging showed preferential localization of ^223^RaCl_2_ to tumor-adjacent bone, with minimal signal in isolated tumor tissue. Immunohistochemistry revealed increased CD4^+^ and CD8α^+^ T-cell infiltration in regions of high γH2AX expression, indicating localized immune modulation. However, combination therapy with ICIs did not enhance tumor control or immune infiltration beyond monotherapy. qPCR demonstrated significant upregulation of Mhc1 only in the combination group, suggesting localized immune activation. Toxicity profiles remained acceptable.

**Conclusions:**

^223^RaCl_2_ localizes primarily to bone surfaces, limiting direct cytotoxic and immunomodulatory effects within the tumor microenvironment. While combination with ICIs did not improve efficacy, these findings provide a platform for studying spatial dose distribution and support future development of tumor-targeted alpha therapies to potentiate immunotherapy in mCRPC.

## 1. Introduction

Prostate cancer is a leading cause of cancer-related morbidity and mortality in men worldwide [1]. It is the second most common malignancy diagnosed in men [2]. While many cases of prostate cancer are indolent, a significant proportion of men are diagnosed with high-risk localized or advanced disease [1,3].

Initially, advanced prostate cancer responds to androgen deprivation therapy [2]. However, the disease inevitably progresses to metastatic castration-resistant prostate cancer (mCRPC) [1,4] which is an incurable stage of the disease [2]. Bone metastases are common in mCRPC, increasing the risk of skeletal-related events that contribute to disease-related morbidity and mortality [5]. Symptoms can include pain, which is associated with reduced survival [6]. The 5-year survival rate for patients with mCRPC is approximately 30% [7].

Radium-223 (^223^RaCl_2_), commercially known as Xofigo (© Bayer AG, Leverkusen, Germany), is a targeted alpha-radiotherapeutic agent that accumulates in areas of high bone turnover, including bone metastases [8]. It is approved for the treatment of mCRPC patients with symptomatic bone metastases and no known visceral disease [9]. It mimics calcium and selectively targets bone, delivering localized alpha radiation to bone metastases, providing increased survival, pain relief and improving quality of life [10]. Completion of the full six-cycle regimen of ^223^RaCl_2_, as well as administration earlier in the treatment course, has been associated with improved overall survival [11,12]. Predictors of successful treatment completion include prior Sipuleucel-T, concurrent denosumab therapy, and lower baseline PSA [13].

Radiation itself is known to stimulate antitumor immunity through mechanisms such as immunogenic cell death and activation of dendritic cells [14]. Importantly, heterogeneous radiation dose distribution within tumors may further amplify immune responses and facilitate checkpoint blockade sensitivity. Low-dose radiation has also been shown to reduce cancer-associated fibroblasts and enhance T-cell infiltration [15]. These immunomodulatory effects support the rationale for exploring low- and intermediate-dose RPT in combination with immunotherapy [15–18]. Notably, tumor-targeted radiopharmaceuticals at low doses have demonstrated curative responses and long-term immune memory in preclinical models, highlighting the potential of RPT to not only debulk tumor but also reshape the tumor-immune landscape for durable response [16–18].

Given the limitations of current therapies and the potential for synergistic effects, there is considerable interest in combining ^223^RaCl_2_ with other treatment modalities, including immunotherapy [19]. The rationale behind this approach lies in the ability of radiation therapy to eradicate tumor cells and modulate the tumor microenvironment, potentially enhancing the efficacy of immune-based treatments [19]. Preclinical data suggests that ^223^RaCl_2_ can induce immunogenic remodeling of the tumor microenvironment [20,21]. However, recent clinical efforts to pair radium with immune checkpoint inhibitors (ICIs) have yielded mixed results. In a randomized phase II study, adding pembrolizumab to ^223^Ra in mCRPC did not meaningfully improve immune infiltration or clinical outcomes versus radium-223 alone, although the regimen was feasible and biologically informative, underscoring the challenge of limited intratumoral deposition when the α-emitter is confined to bone surfaces rather than tumor parenchyma [22]. By contrast, phase 1 study testing a single “priming” dose of ^177^Lu-PSMA-617 followed by pembrolizumab maintenance reported acceptable safety with preliminary activity, and additional trials are exploring whether deeper tumor-localized deposition can better synergize with immunotherapy [23,24].

In this study, we investigate the therapeutic and immunological impact of combining ^223^RaCl_2_ with immune checkpoint inhibitors (ICIs) in a clinically relevant, immunocompetent murine model of prostate cancer bone metastasis. Our hypothesis is that alpha radiation, despite localizing primarily to the tumor-adjacent bone matrix, may still modulate immunogenicity of the tumor microenvironment through localized damage that correlates with the spatial distribution of RT dose. Specifically, we explore whether peripheral deposition of alpha particles around bone-residing tumors is sufficient to elicit immunogenic remodeling that could enhance ICI responsiveness. This work aims to inform the mechanistic underpinnings of clinical observations and to guide further translational investigation of alpha-emitting radiopharmaceutical therapy–immunotherapy combinations.

## 2. Materials and Methods

### 2.1. Cell Lines and Culture

The murine prostate cancer cell line MyC-CaP was obtained from the American Type Culture Collection (ATCC, Manassas, VA, USA; CRL-3255). Cells were transduced with a lentiviral vector encoding luciferase and tdTomato, as previously described [25]. Specifically, pCDH-EF1-Luc-p2a-tdTomato (RRID: Addgene_72486) was used for transduction, followed by sorting for tdTomato fluorescence. Luciferase expression was verified using the Promega Luciferase Assay System (Promega, Madison, WI, USA; Cat. No. QWEE1500). Luciferase-expressing MyC-CaP (MyC-CaP-luc) cells were maintained in Dulbecco’s Modified Eagle Medium (DMEM; Gibco, Grand Island, NY, USA; Cat. No. 11965092) supplemented with 10% fetal bovine serum (FBS; Gibco, Grand Island, NY, USA; Cat. No. A5256701), 100 U/mL penicillin, and 100 μg/mL streptomycin. Cells were cultured under dual antibiotic selection with 6 μg/mL puromycin and 7.5 μg/mL blasticidin to ensure stable luciferase expression. Cell line authentication was performed in accordance with ATCC guidelines based on morphology and growth characteristics. Routine mycoplasma testing using the MycoStrip kit (InvivoGen, San Diego, CA, USA; Cat. No. rep-mys-10) confirmed the absence of contamination.

### 2.2. Murine Tumor Models

All animal studies were approved by the Institutional Animal Care and Use Committee (IACUC) at the University of Wisconsin–Madison (protocol number: M005853) and conducted in accordance with guidelines from the Research Animal Resource Center. The MyC-CaP cell line in FVB mice serves as an immune-competent, mixed osteoblastic and osteolytic model of prostate cancer bone metastasis [26]. Male FVB/N mice (11 weeks old) were purchased from The Jackson Laboratory and allowed to acclimate for seven days prior to study initiation in multi housing of 4 mice per cage.

To minimize surgical discomfort, mice were pre-treated with meloxicam (10 mg/kg, subcutaneously). Anesthesia was induced with isoflurane in an induction chamber and maintained via nose cone throughout the procedure. Mice were positioned supine on a heated surgical surface to maintain normothermia. The right hindlimb was flexed to a 90° angle to visualize the patellar ligament. The surgical area was shaved and aseptically prepped with 70% ethanol. A sterile 27-gauge needle attached to a Luer-tip Hamilton microsyringe was inserted behind the patella and through the patellar ligament into the anterior intercondylar region of the proximal tibia. A total of 5 × 10^4^ MyC-CaP-luc cells suspended in 10 μL of a 1:1 (*v*/*v*) mixture of phosphate-buffered saline (PBS) and Matrigel (Growth Factor Reduced, Corning; Corning, NY, USA) was injected intratibially. Mice were monitored post-operatively and euthanized by CO_2_ inhalation if found moribund, paraplegic, bearing tumors exceeding 1 cm, or at the designated experimental endpoint. Mice were randomized immediately before treatment based on tumor bioluminescence. Only mice with significant bioluminescence of the tumor were included in the study at three weeks. Randomization was performed using the average bioluminescence signal across all enrolled mice as a reference, and mice were assigned such that the mean baseline bioluminescence of each treatment group was comparable to the overall study average. This approach ensured balanced tumor burden across treatment cohorts prior to initiation of therapy. The day of RPT was defined as “day 1” of treatment. Anti-murine CTLA-4 (IgG2c, clone 9D9, NeoClone; Madison, WI, USA) and anti-murine PD-L1 (IgG2b, clone 10F.9G2, BioXCell; Lebanon, NH, USA), 100 μg each, were administered by intraperitoneal injection on days 4, 7, and 10. Due to institutional radiation safety protocols and the use of multiple radioactive isotopes, all investigators were aware of mouse treatment groups.

### 2.3. Radiopharmaceuticals

^223^RaCl_2_ (Xofigo^®^; Bayer HealthCare Pharmaceuticals, Leverkusen, Germany) was calibrated using a dose calibrator (CRC-55tW, Mirion Technologies Inc; Atlanta, GA, USA) prior to administration. Calibration settings were confirmed against a National Institute of Standards and Technology (NIST)-traceable standard at the time of vialing. The empirically determined dial setting was subsequently used for all experimental doses. Radiopharmaceutical solutions were diluted immediately prior to injection using sterile 0.9% sodium chloride. Mice were administered the following activity levels of ^223^RaCl_2_: 400 kBq/kg (0.27 μCi or 270 nCi), 266 kBq/kg (0.18 μCi), 60 kBq/kg (0.04 μCi or 40 nCi), or 10 kBq/kg (7 nCi).

### 2.4. X-Ray Imaging

**Figure 1.**
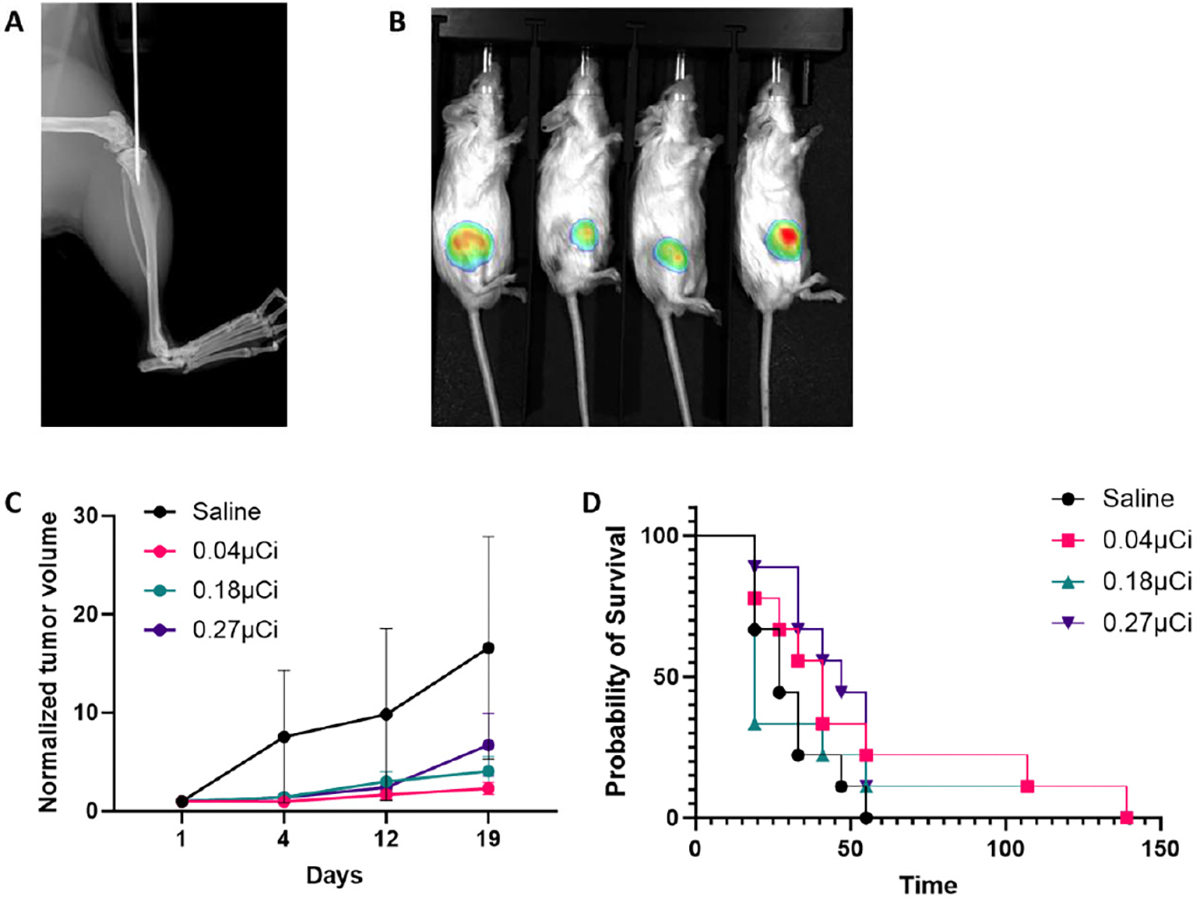
Dose escalation of ^223^RaCl_2_ does not enhance efficacy in a murine model of metastatic prostate cancer. (**A**) Representative X-ray image of intratibial injection of Myc-CaP-luc cells. (**B**) Representative bioluminescence image of mice bearing intratibial Myc-CaP-luc tumors. (**C**) Longitudinal bioluminescence signal (mean ± SEM) in FVB mice (*n* = 9) following treatment with 0.04, 0.18, or 0.27 μCi of ^223^RaCl_2_, or 0.9% NaCl saline control. (**D**) Kaplan–Meier survival analysis of time to humane endpoint in tumor-bearing mice treated with the indicated doses of ^223^RaCl_2_ or saline.

While under 2% isoflurane anesthesia in oxygen, mice were injected with ^223^RaCl_2_ using a Hamilton microliter syringe. Following injection, the syringe was carefully detached to avoid tissue injury, and the mice were promptly transferred to the imaging bed (Figure 1A). X-ray images were acquired using the Faxitron UltraFocus (Tucson, AZ, USA) in vivo imaging system at a spatial resolution of 8 μm. Survival and tumor growth treatment mice were not subjected to x-ray imaging to conserve any radiation dose to the tumors.

### 2.5. Bioluminescence Imaging

Tumor progression in mice (*n* = 10/group) was monitored using in vivo bioluminescence imaging following administration of ^223^RaCl_2_ at doses of 0.27 μCi, 0.18 μCi, or 0.04 μCi. The 0.27 μCi dose was also used in a cohort receiving combinational therapy with immune checkpoint inhibitors (ICIs). Imaging was performed prior to treatment (Day 0) and weekly thereafter. Mice were anesthetized with 2% isoflurane in oxygen, and D-luciferin potassium salt (120 mg/kg; MediLumine, Montreal, QC, Canada) was administered via intraperitoneal injection 7 min prior to image acquisition. Bioluminescence imaging was conducted using the Spectral Instruments Imaging Lago X system (Scott Ireland, ON, Canada). Up to five mice were positioned prone on the imaging bed, separated by physical dividers to prevent signal overlap. Bioluminescence signals were quantified using Aura software v4.5 (Spectral Instruments Imaging; Tucson, AZ, USA), and tumor burden was assessed by normalizing total flux (photons/sec) to each mouse’s Day 0 value.

### 2.6. Micro-CT Imaging

To evaluate bone architecture and tumor-induced lesions, mice were injected with ^223^RaCl_2_ and euthanized at 3, 24, 72, and 168 h post-injection (*n* = 3/time point). Following euthanasia via CO_2_ inhalation, tibiae were harvested and embedded in Tissue-Tek OCT compound (Sakura Finetek) for ex vivo imaging. OCT blocks were placed on a custom dry ice-cooled platform to maintain structural integrity during image acquisition. High-resolution micro-computed tomography (micro-CT) was performed using a MILabs U-SPECT6/CT UHR system (Houten, The Netherlands). Scans were acquired using the UltraFocus Accurate mode with an exposure time of 75 ms, X-ray voltage of 50 kVp, and current of 0.21 mA. Images were reconstructed at 15 μm isotropic voxel resolution using a Hahn projection filter and a Gaussian filter with a 20 μm full-width at half-maximum (FWHM).

### 2.7. Alpha Camera Imaging and Dosimetry

12 mice were injected with 0.27 μCi of ^223^RaCl_2_ (Xofigo), and groups of 3 mice were sacrificed at 3, 24, 72, and 168 h post-injection. Tibiae with metastatic Myc-CaP-luc murine prostate tumor growth were harvested, embedded in OCT, and immediately frozen. Frozen samples were sectioned laterally using a Leica CM1950 cryostat, with emphasis on tumor-infiltrated regions. Sections for iQID imaging were cut at 30 μm thickness, except for the 168 h time point, which used 50 μm sections. Tissue sections were mounted on charged glass slides (Fisherbrand Superfrost Plus^®^; Waltham MA, USA) for both iQID and histological imaging.

Sections were placed in the scanner bed beneath a thin sheet of mylar (Ludlum Measurements 01–5859; Sweetwater, TX, USA) and ZnS:Ag scintillating phosphor screen (Eljen Technology EJ-440) for alpha detection. One scan per time point was performed, with each scan including three serial sections. Scan durations varied: 18 h for 3 h and intended 72 h time points, 16 h for 24- and 168 h time points. The 72 h scan was terminated early at 5.3 h due to acquisition error.

Shadowgraph images were acquired using a phosphorescent disk to generate transmitted light silhouettes of the tissue, which were imported into ImageJ v1.54p for registration. After H&E staining, histological images were captured using an Infinity2–2M monochrome camera on an Olympus CKX41 inverted microscope. A 2× objective was used for most images, except for two sections from the 3 h time point, which used 4× magnification for improved resolution.

Histological images were co-registered to shadowgraphs using SIFT feature extraction and rigid transformation in ImageJ. Final alignment was refined using the bunWarpJ plugin v2.6.13 for deformable registration. Background removal and mask generation were performed in Adobe Photoshop, followed by creation of binary masks representing the tissue silhouette. These masks were applied in ImageJ using the “Image Calculator” with an AND operation to filter out non-tissue pixels from the iQID count rate maps. The registered histological images were then overlaid on the filtered iQID images to produce aligned autoradiographic-histology composites.

The iQID count rate was converted to a Bq/mL activity concentration map in AMIRA software v2020.3 (AMIRA, Mercury Computer Systems, Berlin, Germany) using a previously established cps-to-Bq calibration curve specific to ^223^Ra. Each tissue section’s activity concentration was co-registered with a liquid water density map at 0.02 mm × 0.02 mm in-plane resolution and the sliced section thickness (0.03 and 0.05 mm). Tumor ROIs were manually segmented based on co-registered H&E-stained histological images and mapped to the corresponding liquid water volume. Absorbed dose rates at each time point were computed using the RAPID platform, an in-house Monte Carlo simulation dosimetry framework based on Geant4. Absorbed dose rates within the ROIs across four time points were fit to a mono-exponential decay model, and the resulting decay parameters were used to compute the time-integrated absorbed dose for each tissue section.

### 2.8. Immunohistochemistry and Histology

Serial sections of tumor-bearing tibiae prepared for alpha camera imaging were airdried and rinsed in phosphate-buffered saline (PBS). Slides designated for histological analysis were stained with eosin and counterstained with hematoxylin. For immunohistochemistry (IHC), OCT compound was carefully trimmed from the frozen tissue, and tibiae were thawed in 10% neutral buffered formalin. After complete thawing, formalin was refreshed to remove residual OCT, and samples were fixed for 48 h. Decalcification was performed in 0.25 M EDTA at 4 °C with multiple solution changes over a 3-week period. Following decalcification, tissues were rinsed and stored in 70% ethanol until paraffin embedding. Paraffin-embedded sections were deparaffinized and rehydrated using standard protocols. Antigen retrieval was performed using Antigen Unmasking Solution, pH 9.0 (Vector Laboratories; Newark, CA, USA), in a Biocare Medical Decloaking Chamber. Sections were washed in Tris-buffered saline (TBS), then incubated in 3% hydrogen peroxide to quench endogenous peroxidase activity. Blocking was performed using 10% goat serum in TBS containing 0.1% Tween-20 (TBT). Slides were incubated with primary antibodies (listed in Table S1) overnight at 4 °C on an orbital shaker. Following primary incubation, slides were washed and incubated with an HRP-conjugated secondary antibody using the ImmPRESS detection system (Vector Laboratories) for 1 h at room temperature. Immunoreactivity was visualized with DAB Substrate Kit (Cell Signaling Technology;. Danvers, MA, USA), followed by counterstaining with Mayer’s hematoxylin (Sigma-Aldrich; Burlington, MA, USA) and mounting. At least three 20× fields per section were quantified for positive staining by a blinded observer. Tumor regions were identified based on histological landmarks and validated by co-localization with physiological markers.

### 2.9. Gene Expression Analysis

Tumor-bearing tibiae were carefully bisected using sterile surgical instruments, and tumor tissue was gently scraped from the bone marrow cavity. Harvested tissues were immediately homogenized in TRIzol Reagent (Thermo Fisher Scientific; Waltham, MA, USA) using a Bead Ruptor Elite bead mill homogenizer (Omni International; Nennesaw, GA, USA). Complementary DNA (cDNA) was synthesized using the QuantiTect Reverse Transcription Kit (QIAGEN; Venlo, Netherlands). Quantitative PCR (qRT-PCR) was performed using TaqMan Fast Advanced qPCR Master Mix (Thermo Fisher Scientific; Waltham, MA, USA). Thermal cycling conditions on the QuantStudio 6 (Applied Biosystems; Foster City, CA, USA) were as follows: UDG activation at 50 °C for 2 min, Dual-Lock DNA polymerase activation stage at 95 °C for 2 min, then 40 PCR cycles—denaturation at 95 °C for 1 s and annealing/extension at 60 °C for 20 s. Ct values were exported to Excel, and fold changes normalized to untreated controls were calculated using the ∆∆Ct method. 18s served as endogenous controls. A complete list of TaqMan probes is provided in Table S2. All qPCR experiments were performed in duplicate and presented as aggregate data.

### 2.10. Ex Vivo Biodistribution

To assess tissue-level distribution of ^223^RaCl_2_, mice were injected intravenously with 9.99 kBq (0.27 μCi) and euthanized at 3, 24, 72, and 168 h post-injection (*n* = 4/time point). Following euthanasia by CO_2_ inhalation, organs including heart, lungs, liver, spleen, pancreas, kidneys, stomach, intestines, bone, muscle, skin, blood, brain, and tumor-bearing tibiae were harvested, blotted dry, and weighed. Tumor-bearing tibiae were carefully bisected using sterile surgical tools, and intramedullary tumor tissue was gently scraped from the marrow cavity prior to weighing and analysis to differentiate tumor-associated activity from surrounding bone. Radioactivity was quantified using a gamma counter (PerkinElmer Wizard^2^ or Hidex AMG; Westham, MA, USA or Turku, Finland, respectively). Data were decay-corrected to the time of injection and expressed as percent injected activity per gram of tissue (%IA/g) (Figure S2).

### 2.11. Toxicity Assessments

Toxicity was evaluated through comprehensive metabolic panel (CMP) and complete blood count (CBC) analyses. Mice were anesthetized and 700 μL of whole blood was collected via terminal intracardiac puncture. Blood for CBC analysis was collected into EDTA-coated tubes and analyzed using a VetScan HM5 hematology analyzer (Abaxis, Union City, CA, USA). For serum biochemistry, blood was collected in serum separator tubes and centrifuged at 4000 rpm for 10 min. Serum was either analyzed immediately or stored at −20 °C and thawed on ice prior to analysis. CMPs were performed using the Abaxis VetScan VS2 analyzer (Abaxis, Union City, CA, USA). Measured parameters included markers of liver function (ALT, AST, ALP, total bilirubin), renal function (BUN, creatinine), electrolytes, glucose, and total protein. CBC included assessments of white blood cells, red blood cells, hemoglobin, lymphocytes, and platelet counts (Figure S1).

### 2.12. Statistical Analysis

All statistical analyses were conducted using Graphpad Prism v10.1.1 and R v4.4.2. Two-way ANOVA or multiple comparison tests using Tukey’s honestly significant difference (HSD) test were applied to assess group differences in gene expression and immunohistochemistry data. Results from each mouse were summarized by the time-weighted average (area under the volume-time curve, calculated using trapezoidal method). Time-weighted averages were compared between treatment groups overall by a Kruskal–Wallis test. Survival curves compared with a log rank test. For tumor growth and survival graphs, *, *p* < 0.03; **, *p* < 0.002; and ***, *p* < 0.001. For gene expression analysis and immunohistochemistry graphs, *, *p* < 0.03; **, *p* < 0.0021; ***, *p* < 0.0002; and ****, *p* < 0.0001.

## 3. Results

### 3.1. Dose Escalation of ^223^RaCl_2_ Does Not Enhance Efficacy in a Murine Model of Metastatic Prostate Cancer

Several approaches exist for establishing orthotopic pseudo-mCRPC models in mice, including ventral caudal artery, intraosseous, and intracardiac injection [27]. Among these, intraosseous injection provides precise localization of tumor cells to the bone, enables a relevant immune microenvironment not achievable in subcutaneous models, and allows for consistent and reproducible tumor establishment [28]. We utilized an established immunocompetent model of MyC-CaP prostate cancer in FVB mice [26] modified to express luciferase (MyC-CaP-luc) for in vivo monitoring. Tumors were introduced via intratibial injection (Figure 1A), and luciferase expression enabled longitudinal bioluminescence imaging (Figure 1B). Mice were treated with escalating doses of ^223^RaCl_2_ (0.04 μCi, 0.18 μCi, and 0.27 μCi). Mice did not exhibit acute toxicity effects (Figure S1). All treatment groups exhibited delayed tumor progression and modest survival prolongation compared to the saline control; however, these differences were not statistically significant, with a *p*-value of 0.630 for time-weighted average tumor burden according to Kruskal–Wallis test, and a *p*-value of 0.294 by log-rank test for survival from treatment initiation (Figures 1C,D and S3A). These results indicate that increasing the dose of ^223^RaCl_2_ does not enhance tumor growth control in this model. This may be consistent with clinical findings where ^223^RaCl_2_ monotherapy yields survival benefit without significant tumor regression [29,30].

### 3.2. Co-Localization of ^223^RaCl_2_ Uptake with Metastatic Tumor Directly Effects the Tumor-Absorbed Dose

**Figure 2.**
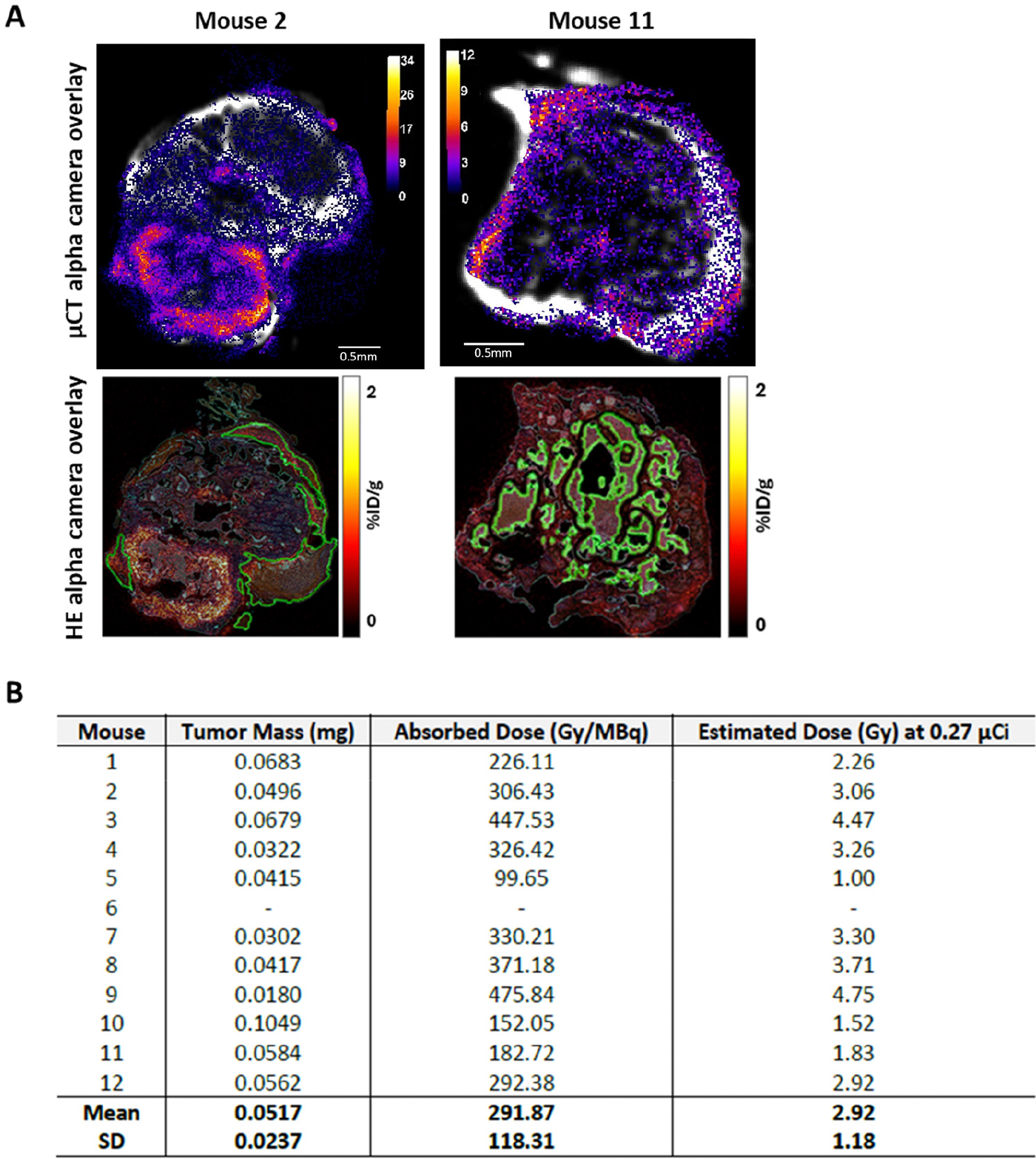
Co-localization of ^223^RaCl_2_ uptake and the metastatic tumor directly affects the absorbed dose to the tumor. (**A**) Representative axial images of tibiae injected with Myc-CaP-luc cells, including micro-CT, α-camera overlay, and hematoxylin and eosin (H&E) sections with corresponding α-camera overlays. Tumor regions are outlined in green. (**B**) Table summarizing mean tumor masses and absorbed dose estimates following ^223^RaCl_2_ administration. The mean absorbed dose was 10.8 Gy/μCi (Note: tumor region information was not obtainable for mouse 6).

The therapeutic mechanism of ^223^RaCl_2_ relies on its calcium-mimetic properties, allowing preferential uptake at sites of active bone remodeling—osteoblastic activity being a hallmark of mCRPC in patients [29,31,32]. However, this bone-targeted localization limits direct penetration of radiation into the islands of tumor cells within the tumor microenvironment. In our study, gamma counter measurements revealed that while the tumor-bearing tibiae exhibited elevated radioactivity, the isolated tumor tissue extracted from the bone marrow cavity showed minimal to no detectable ^223^RaCl_2_ signal (Figure S2). To address this observation and more accurately determine the tumor-absorbed dose, we implemented a multimodal microdosimetry approach. Tibiae with established MyC-CaP-luc tumors were analyzed using high-resolution micro-CT, alpha camera imaging, and hematoxylin and eosin (H&E) staining to map ^223^RaCl_2_ distribution in the bone and confirm tumor morphology (Figure 2A). Based on tumor volume and absorbed dose per administered activity (Gy/MBq) from alpha camera images, we estimated the absolute absorbed dose to tumor tissue for each mouse (Figure 2B). Although the administered activity of radium-223 was 0.27 μCi, microdosimetric analysis estimated the absorbed tumor dose to range from 1.0 to 4.75 Gy, with a mean of 2.92 Gy ± 1.18 Gy. Importantly, we observed that tumors in regions co-localizing with ^223^RaCl_2_ deposition received higher absorbed doses, indicating that spatial overlap between ^223^RaCl_2_ uptake and tumor could be a key determinant of therapeutic efficacy.

### 3.3. Histological Assessment of the Immunological Effects of ^223^RaCl_2_ Absorbed Dose in Established Bone Metastases

**Figure 3.**
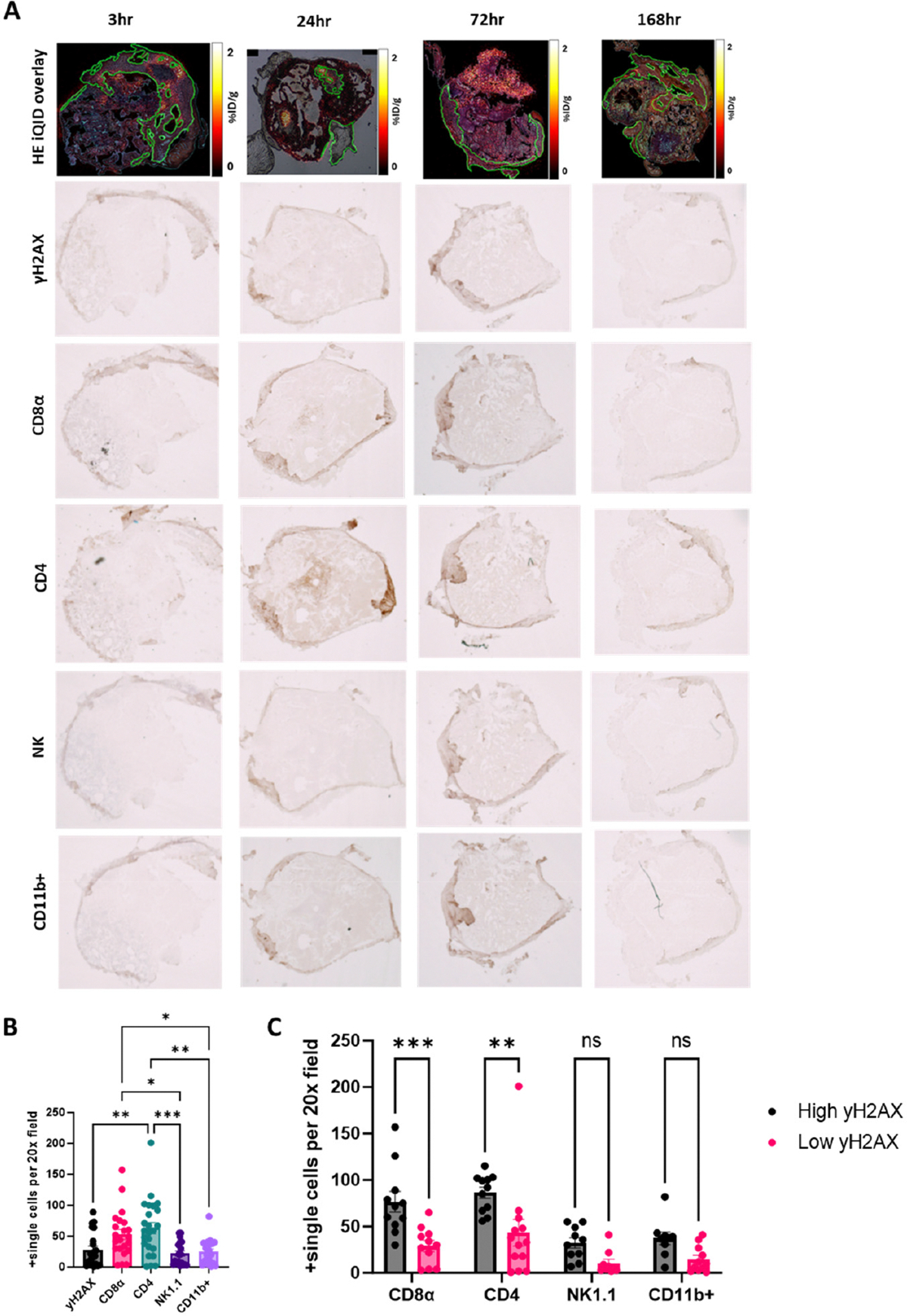
Histological assessment of the immunological effects of absorbed dose of ^223^RaCl_2_ in established bone metastases. (**A**) Representative overlays of hematoxylin and eosin (H&E) staining with α-camera imaging, alongside serial-sectioned immunohistochemistry for DNA damage (γH2AX), T cells (CD8α, CD4), natural killer (NK) cells, and myeloid cells (CD11b+) following treatment with 0.27 μCi of ^223^RaCl_2_ at 3, 24, 72, and 168 h post-injection. (**B**) Quantification of positively stained cells per 20× field, presented as mean ± SEM. (**C**) Immune cell counts stratified by regions with high versus low γH2AX expression to assess spatial correlation of DNA damage with immune infiltration. *, *p* < 0.03; **, *p* < 0.0021; and *** *p* < 0.0002.

To evaluate the immune response induced by ^223^RaCl_2_ in bone metastatic lesions, tibiae previously imaged by alpha camera and micro-CT were serially sectioned and subjected to immunohistochemical (IHC) staining. Markers for immune cell infiltration included CD8α (cytotoxic T cells), CD4 (helper T cells), NK1.1 (natural killer cells), and CD11b (myeloid cells). γH2AX staining was used to identify regions of DNA double-strand breaks, indicative of localized alpha radiation damage (Figure 3A). IHC staining was evaluated using brightfield microscopy at 20× magnification. For each tissue section, representative fields of tumor were selected to avoid areas of necrosis, tissue folds, or staining artifacts. A minimum of 3–5 non-overlapping fields per section were analyzed. Positive cells were manually counted by an independent evaluator who was blinded to the experimental staining consistent with marker localization. Quantitative analysis showed that CD8α^+^ and CD4^+^ T cells were significantly more abundant within the tumor microenvironment compared to NK1.1^+^ and CD11b^+^ cells. Specifically, CD8α^+^ T cell infiltration was significantly greater than NK1.1^+^ (*p* < 0.03) and CD11b^+^ (*p* < 0.05) cell populations. Similarly, CD4^+^ T cells were more abundant than γH2AX^+^ (*p* < 0.004), NK1.1^+^ (*p* < 0.001), and CD11b^+^ (*p* < 0.002) cells (Figure 3B). To examine the spatial relationship between radiation-induced DNA damage and immune infiltration, tissue sections were stratified into regions of high versus low γH2AX expression. The γH2AX-based spatial stratification was performed by correlating serial tissue sections and classifying them according to DNA damage burden. Sections were stratified into low and high γH2AX groups based on whether the number of γH2AX-positive cells fell below or above the overall mean γH2AX-positive cell count across all analyzed sections. This approach enabled spatially resolved comparison of biological features relative to the extent of radiation-induced DNA damage. In regions with high γH2AX staining, CD8α^+^ (*p* < 0.001) and CD4^+^ (*p* < 0.002) T cell counts were significantly elevated compared to low γH2AX regions (Figure 3C). These findings suggest that T cell infiltration is enhanced in areas of greater radium-induced damage, supporting a role for localized alpha particle exposure in driving immunogenic remodeling of the tumor microenvironment [16,20,21].

### 3.4. Combination of ^223^RaCl_2_ and ICIs Does Not Enhance Efficacy in a Murine Model of Metastatic Prostate Cancer

**Figure 4.**
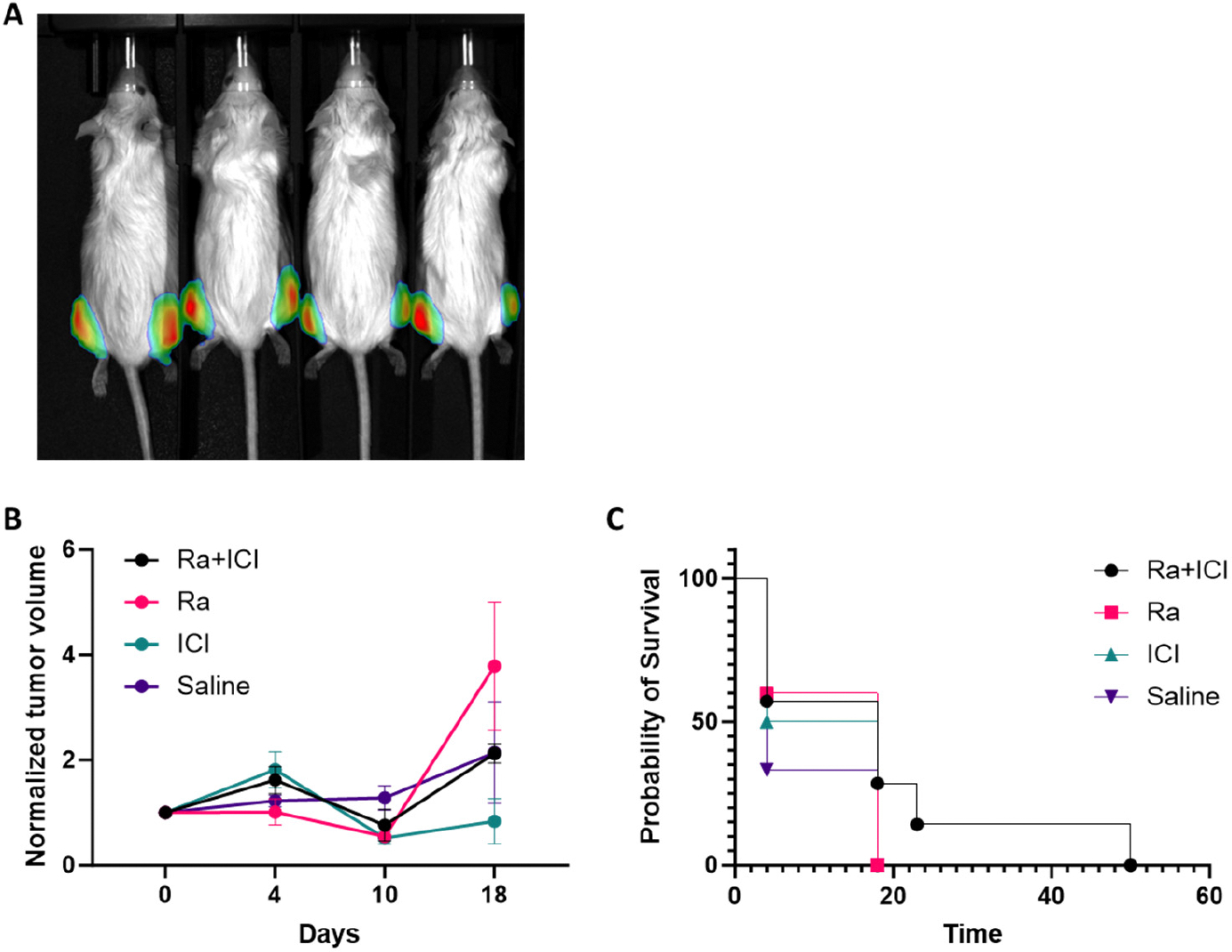
Combination of ^223^RaCl_2_ and ICIs does not enhance efficacy in a murine model of metastatic prostate cancer. (**A**) Representative bioluminescence image of mice bearing bilateral intratibial Myc-CaP-luc tumors. (**B**) Longitudinal bioluminescence signal (mean ± SEM) in FVB mice (*n* = 9) following treatment with 0.27 μCi of ^223^RaCl_2_, 0.27 μCi of ^223^RaCl_2_ combined with ICIs (anti-PD-L1 and anti-CTLA-4), ICIs alone, or 0.9% NaCl saline control. (**C**) Kaplan–Meier survival analysis of time to humane endpoint in tumor-bearing mice treated with the indicated regimens.

Building on our previous work demonstrating the immunostimulatory potential of combining alpha and beta tumor targeting radiopharmaceutical therapy (RPT) with immune checkpoint inhibitors (ICIs) [16–18], we investigated this combination in the MyC-CaP-luc metastatic prostate cancer model. To increase statistical power and tumor burden for evaluation, MyC-CaP-luc cells were injected into both tibiae of each mouse (Figure 4A). Mice received either ^223^RaCl_2_ monotherapy or combination therapy with ICIs (anti-CTLA-4 and anti-PD-L1). However, neither monotherapy nor combination therapy demonstrated a statistically significant improvement in tumor growth inhibition (*p* = 0.396, Kruskal–Wallis test) or overall survival (*p* = 0.481, log-rank test) compared to the controls (Figures 4B,C and S3B). These results suggest that, in this model, combining ICIs with radium-223 does not enhance therapeutic efficacy, potentially due to the limited radiation penetration into the tumor cell islands in the tumor microenvironment.

### 3.5. Histological and qPCR Assessments of the Immunological Effects of ^223^RaCl_2_ and ICIs in Established Bone Metastases

**Figure 5.**
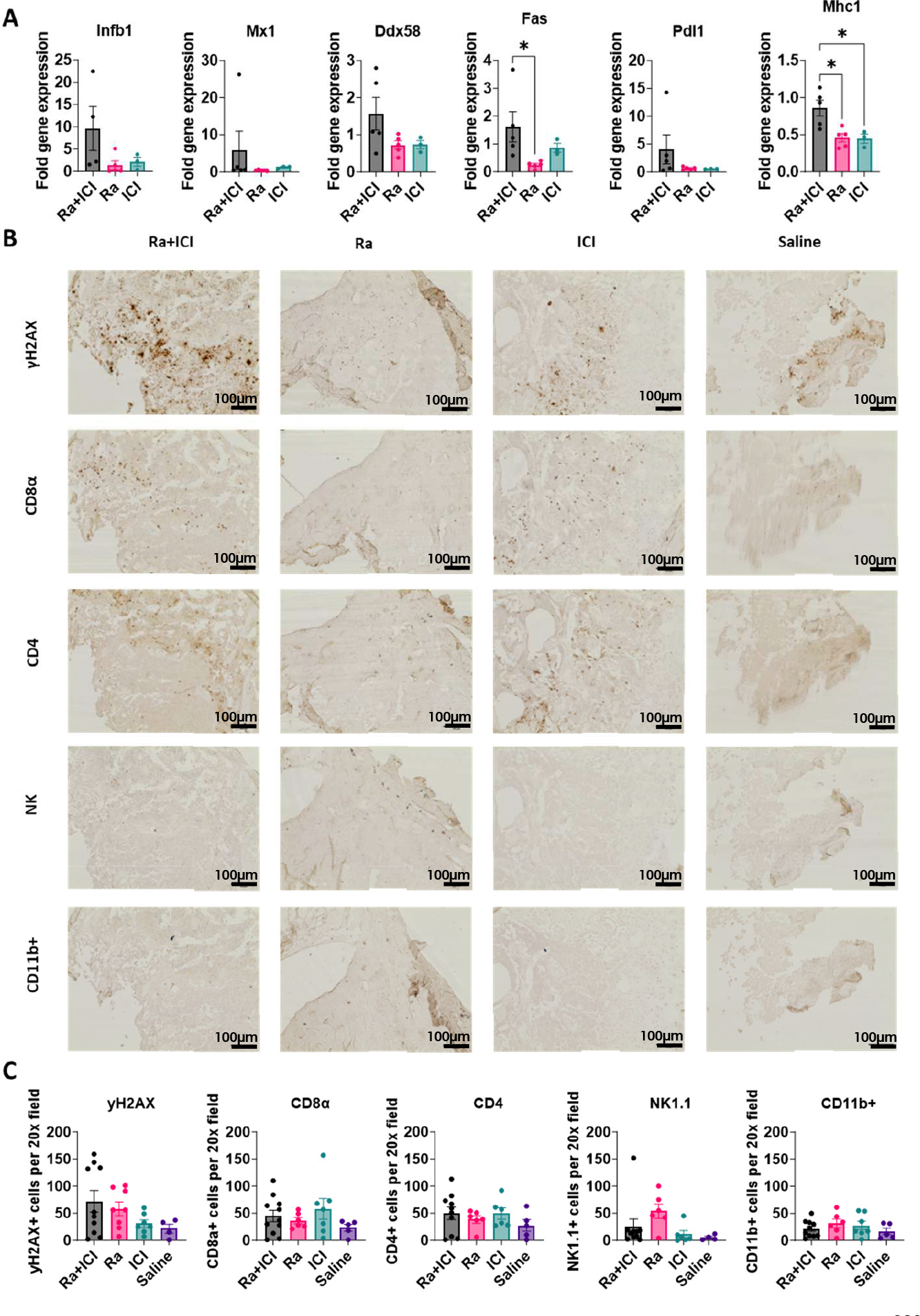
Histological and qPCR assessments of the immunological effects of ^223^RaCl_2_ and ICIs in established bone metastases. (**A**) qPCR analysis of tumor tissue from bilateral intratibial Myc-CaP-luc tumors showing treatment-dependent upregulation of Fas and Mhc1 expression. (**B**) Representative immunohistochemistry at 20× magnification for DNA damage (γH2AX), CD8^+^ T cells, CD4^+^ T cells, NK cells, and myeloid cells (CD11b^+^). (**C**) Quantification of positively stained cells per 20× field, presented as mean ± SEM. *, *p* < 0.03.

To evaluate the immunological effects of ^223^RaCl_2_ and ICIs within the tumor microenvironment, tibiae were harvested on Day 14 post-treatment. One tibia from each animal was surgically bisected, and intramedullary tumor tissue was scraped from the bone marrow cavity for RNA extraction and real-time quantitative PCR (qRT-PCR) analysis (Figure 5A). Although *Ifnb1* expression was elevated in the combination treatment group (Ra + ICI) compared to either monotherapy group (Ra: *p* = 0.15; ICI: *p* = 0.27), these differences did not reach statistical significance. In contrast, *Mhc1* expression was significantly upregulated only in the combination therapy group compared to both ^223^RaCl_2_ alone (*p* < 0.02) and ICI alone (*p* < 0.03). The contralateral tibiae were formalin-fixed, paraffin-embedded (FFPE), and processed for immunohistochemical staining (Figure 5B). Quantitative analysis revealed no significant differences between the Ra + ICI and monotherapy groups in the infiltration of CD8α^+^ T cells, CD4^+^ T cells, NK1.1^+^ natural killer cells, or CD11b^+^ myeloid cells (Figure 5C).

## 4. Discussion

We and others have shown that the low dose tumor targeted RPT can produce curative results with T cell memory induction, when used in combination with immune checkpoint inhibitors in a variety of solid tumor types in syngeneic mouse models [16–18].

The limited penetration radiation from radium-223 into the tumor cell islands within tumor microenvironment, of bone metastases models, owing to its mechanism of uptake as a calcium mimetic that preferentially targets peri tumoral bone remodeling sites [29,31–33], poses a potential limitation to its therapeutic efficacy when used in combination with immunotherapies.

A barrier to translational progress is the lack of standardized, immunocompetent murine models for mCRPC bone metastases that permit rigorous evaluation of therapeutic efficacy, immune activation, and survival. In this study, we established a clinically relevant, immune-competent bone metastasis model of prostate cancer. Using this system, we found that ^223^RaCl_2_ localized predominantly to bone surfaces adjacent to tumor tissue, resulting in limited intratumoral penetration and a spatially restricted immunomodulatory effect. Although tumor-bearing tibiae exhibited high overall uptake of ^223^RaCl_2_, signal from isolated tumor tissue was minimal (Figure S2). Microdosimetric estimates further highlighted this limitation: while 0.27 μCi was administered, absorbed tumor doses ranged from 1.0 to 4.75 Gy (mean 2.92 ± 1.18 Gy), reflecting steep dose gradients at the bone–tumor interface and insufficient irradiation of the tumor bulk. This distribution pattern likely restricts direct cytotoxicity and limits the capacity of ^223^RaCl_2_ to induce immunogenic cell death, which may explain the lack of synergy with immune checkpoint inhibitors (Figure 5). An important consideration in interpreting these findings is the heterogeneous nature of radiation dose delivery within the bone tumor microenvironment following ^223^RaCl_2_ administration. Alpha-emitting radiopharmaceuticals generate steep dose gradients over micrometer-scale distances, resulting in regions of high local energy deposition adjacent to bone surfaces and substantially lower doses within tumor compartments distal to sites of radionuclide localization [34,35]. While such focal irradiation is sufficient to induce DNA damage and localized immune modulation, it may fall below the threshold required for widespread immunogenic cell death across the tumor mass [34,36,37]. This spatially constrained dose distribution likely limits the release of tumor-associated antigens and danger signals necessary to fully engage dendritic cell priming and downstream T-cell mediated antitumor immunity [38–40]. Within the bone microenvironment, where immune accessibility is already tightly regulated, these microdosimetric effects may further restrict the capacity of immune checkpoint inhibitors to amplify antitumor responses. Importantly, these findings align with prior reports and reinforce the need to consider spatial dose heterogeneity when designing combination strategies [10,41]. In the context of mCRPC, where treatment often integrates abiraterone, enzalutamide, docetaxel, cabazitaxel, sipuleucel-T, and radium-223, a nuanced understanding of each agent’s mechanism of action and interaction is critical for advancing effective therapeutic combinations [42]. Integrating radiation dose heterogeneity with biological readouts of immune activation provides a framework for distinguishing model-specific spatial limitations of ^223^RaCl_2_ from broader therapeutic principles that may be addressed through tumor-targeted alpha emitters or optimized combination strategies.

Several limitations should be acknowledged on the model. First, these studies were conducted exclusively in mice, and the immune system, bone physiology, and tumor–bone interactions may not fully recapitulate those in humans. Second, the spatial relationships between tumor cells, bone surfaces, and radionuclide deposition are likely influenced by tumor size and architecture; it remains unclear whether the same distribution patterns observed here would apply to micrometastatic disease or to the more heterogeneous tumor burden seen in patients. Third, our experiments evaluated a single cycle of ^223^RaCl_2_, whereas clinical treatment involves multiple cycles administered over time. In patients, dynamic bone remodeling during repeated dosing may alter the spatial delivery of radiation to tumor cell clusters, potentially changing both efficacy and immune modulation. Lastly, an important translation limitation of this study is that only a single cycle of radium-223 was administered, whereas clinical treatment regimens involve multiple repeated cycles, which may produce cumulative radiobiological and immunomodulatory effects not captured in this study. These factors highlight the need for cautious interpretation when extrapolating preclinical findings to the clinical setting.

Future investigations could focus on enhancing the spatial and mechanistic characterization of ^223^RaCl_2_ activity within the bone tumor microenvironment to better inform precision radiopharmaceutical-immunotherapy strategies. Because ^223^RaCl_2_ acts as a calcium mimetic and preferentially accumulates at sites of active bone modeling, elucidating the spatial relationship between calcium deposition and tumor localization remains an important next step. Incorporation of calcium-sensitive histological methods, such as von Kossa staining, would strengthen interpretation of biodistribution and target specificity. Additionally, quantitative correlation of radionuclide localization with DNA damage such as γH2AX would provide direct evidence linking heterogeneous microdosimetric exposure to functional tumor response. While the present study integrates μCT, alpha camera imaging, and γH2AX staining to demonstrate spatially restricted biological effects, further optimization of IHC analyses on undecalcified serial bone sections would enable more precise co-registration of bone remodeling, radiopharmaceutical deposition, and tumor response. In addition, comprehensive immunophenotypic analyses using flow cytometry of peripheral immune cells from blood, spleen, and tumor tissue across monotherapy and combination treatment groups would be highly informative for delineating systemic versus intratumoral immune modulation. In the current study, however, the early experimental timepoint and confinement of tumor cells within the intramedullary space limited recovery of sufficient tumor material for flow cytometric analysis. Allowing tumors to progress to larger volumes and extend beyond the intramedullary compartment, or increasing the sample size and pooling the tumor cells, would facilitate robust tumor cell isolation and enable definitive immunophenotypic comparisons, thereby strengthening mechanistic interpretation beyond phenotypic observation.

The therapeutic potential of tumor cell targeted alpha-emitting radiopharmaceuticals in combination with immunotherapies remains promising. Alpha therapies with tumor-targeting mechanisms, may overcome the spatial barriers observed in our model by delivering radiation more homogeneously and directly to tumor cells. As such, future work should explore the integration of tumor cell targeted alpha emitters with immune checkpoint blockade to evaluate the potential of combination alpha-emitting radiopharmaceutical-immunotherapy strategies in mCRPC [43].

## 5. Conclusions

This study establishes an immunocompetent murine model of prostate cancer bone metastasis using intratibial injection of MyC-CaP-luc cells in syngeneic FVB mice, providing a robust platform to investigate combination strategies involving alpha-emitting radiopharmaceuticals and immunotherapy. Our results demonstrate that while radium-223 (^223^RaCl_2_) preferentially accumulates in tumor-bearing tibiae, its localization remains confined to the bone matrix, with minimal penetration into tumor cells within the tumor microenvironment. This spatial distribution, dictated by ^223^Ra’s calcium-mimetic properties and affinity for areas of active bone remodeling, underlies the observed lack of direct antitumor efficacy in this model when combined with immune checkpoint inhibitors (ICIs).

From a microdosimetric perspective, alpha particles deliver high linear energy transfer (LET) radiation over short ranges (~50–100 μm), resulting in highly localized cytotoxicity. This creates steep dose gradients in the tumor microenvironment, with high radiation doses at the periosteal bone surfaces but a rapid falloff within adjacent soft tissue. This heterogeneity in dose deposition may have dual effects: while it limits widespread tumor cell kill, it may also generate highly immunogenic zones of damage, potentially sufficient to initiate immune activation under the right conditions. Our findings suggest that this peripheral irradiation—although not curative—may provide an immune-stimulating effect, as evidenced by localized T cell recruitment and gene expression changes.

In addition, our study revealed evidence of localized immune stimulation. Immunohistochemical analysis showed increased infiltration of CD4^+^ and CD8α^+^ T cells in regions with high γH2AX expression—suggesting that focal radiation-induced DNA damage may serve as a stimulus for immune cell recruitment. Furthermore, qPCR analysis revealed upregulation of Mhc1 gene expression in the combination therapy group.

Although these immune effects did not translate into significant tumor control or survival benefit in the combinational therapy approach compared to monotherapy, they align with preclinical and clinical observations that alpha radiation can modulate tumor immunity. Prior studies using tumor-targeted alpha emitters have demonstrated curative responses and immune memory induction at low doses, emphasizing the importance of intratumoral deposition for maximizing both cytotoxic and immunostimulatory effects. In contrast, ^223^RaCl_2_’s efficacy in mCRPC appears to derive primarily from its impact on the bone microenvironment rather than direct tumor targeting in our model.

The model developed in this study enables systematic investigation of radiopharmaceutical biodistribution, immune responses, and toxicity in an immunocompetent setting that closely mirrors the clinical disease. The integration of bioluminescence imaging, micro-CT, alpha-camera imaging, histology, and gene expression profiling provides a comprehensive toolkit for future mechanistic and therapeutic investigations. These capabilities are particularly valuable for evaluating next-generation alpha-emitting agents or combination regimens aimed at enhancing intratumoral delivery and immune engagement.

Looking forward, identifying optimal dosing and sequencing with ICIs may help harness the immunomodulatory potential of ^223^RaCl_2_ in a clinically meaningful way. Ongoing clinical trials investigating combinations of radium-223 with enzalutamide, pembrolizumab, and other agents in mCRPC support the translational relevance of this work.

In summary, this study highlights the utility of an immunocompetent prostate cancer bone metastasis model to evaluate combination alpha-RPT and immunotherapy approaches. While ^223^RaCl_2_ primarily localizes to the tumor periphery in bone, its ability to modulate the immune microenvironment warrants further exploration, particularly in the context of rationally designed combination strategies. These findings contribute to the growing understanding of how alpha radiation may synergize with immunotherapy and support the continued development of radiopharmaceutical–immune combinations in advanced prostate cancer.

## Supplementary Material

Supplementary Tables & Figures

The following supporting information can be downloaded at: https://www.mdpi.com/article/10.3390/precisoncol1010005/s1, Figure S1: Acute toxicity profile of ^223^RaCl_2_; Figure S2: Biodistribution of ^223^RaCl_2_ in tumor bearing tibiae and isolated tumor compared to background; Figure S3: Statistical tests done on survival and tumor growth curves; Table S1: List of antibody utilized for IHC; Table S2: List of TaqMan probes utilized for quantitative RT-PCR experiments.

## Data Availability

The original contributions presented in this study are included in the article/Supplementary Materials. Further inquiries can be directed to the corresponding author.

## Abbreviations

The following abbreviations are used in this manuscript:

^223^RaCl_2_: Radium-223 dichloride
mCRPC: Metastatic castration-resistant prostate cancer
ICIs: Immune checkpoint inhibitors
MyC-CaP: Myc-induced c-Myc-driven Prostate Cancer cell line (murine)
Luc: Luciferase
MyC-CaP-luc: Luciferase-expressing MyC-CaP prostate cancer cells
μCi/kBq: Microcurie/Kilobecquerel
CTLA-4: Cytotoxic T-lymphocyte-associated protein 4
PD-L1: Programmed death-ligand 1
CD4+: Cluster of Differentiated 4 positive T cells
CD8α+: Cluster of Differentiation 8 alpha-positive T cells
γH2AX: Phosphorylated histone H2A.X
MHC I: Major Histocompatibility Complex Class I
RPT: Radiopharmaceutical therapy
FDA: Food and Drug Administration
ATCC: American Type Culture Collection
DMEM: Dulbecco’s Modified Eagle Medium
FBS: Fetal Bovine Serum
PBS: Phosphate-buffered saline
IACUC: Institutional Animal Care and Use Committee
NIST: National Institute of Standards and Technology
OCT: Optimal Cutting Temperature compound
H&E: Hematoxylin and eosin
IHC: Immunohistochemistry
HRP: Horseradish peroxidase
DAB: 3,3′-Diaminobenzidine
TBS: Tris-buffered saline
TBT: Tween Tris-buffered saline
EDTA: Ethylenediaminetetraacetic acid
cDNA: Complementary DNA
qPCR/qRT-PCR: Quantitative real-time polymerase chain reaction
UDG: Uracil-DNA glycosylase
Ct: Cycle threshold
%IA/g: Percent injected activity per gram of tissue
CMP: Comprehensive metabolic panel
CBC: Complete blood count
ALT: Alanine aminotransferase
AST: Aspartate aminotransferase
BUN: Blood urea nitrogen
ROI: Region of interest
FWHM: Full width at half maximum
NCI: National Cancer Institute
ANOVA: Analysis of variance
HSD: Honestly Significant Difference (Tukey test)
SEM: Standard error of the mean
PSMA: Prostate-specific membrane antigen
^177^Lu: Lutetium-177
RAPID: Radiopharmaceutical Imaging and Dosimetry platform
Geant4: Geometry and Tracking
