# Supplementary material for: Preclinical Evaluation of Radium-223 and Immune Checkpoint Inhibitors Using an Immune-Competent Model of Prostate Cancer Bone Metastases
Source: Precis Oncol. Author manuscript; Available in PMC 2026 Apr 30. (PMC13127722; doi:10.3390/precisoncol1010005)
Supplement: Supplementary Tables & Figures [file NIHMS2153740-supplement-Supplementary_Tables___Figures.pptx]

## Slide 1
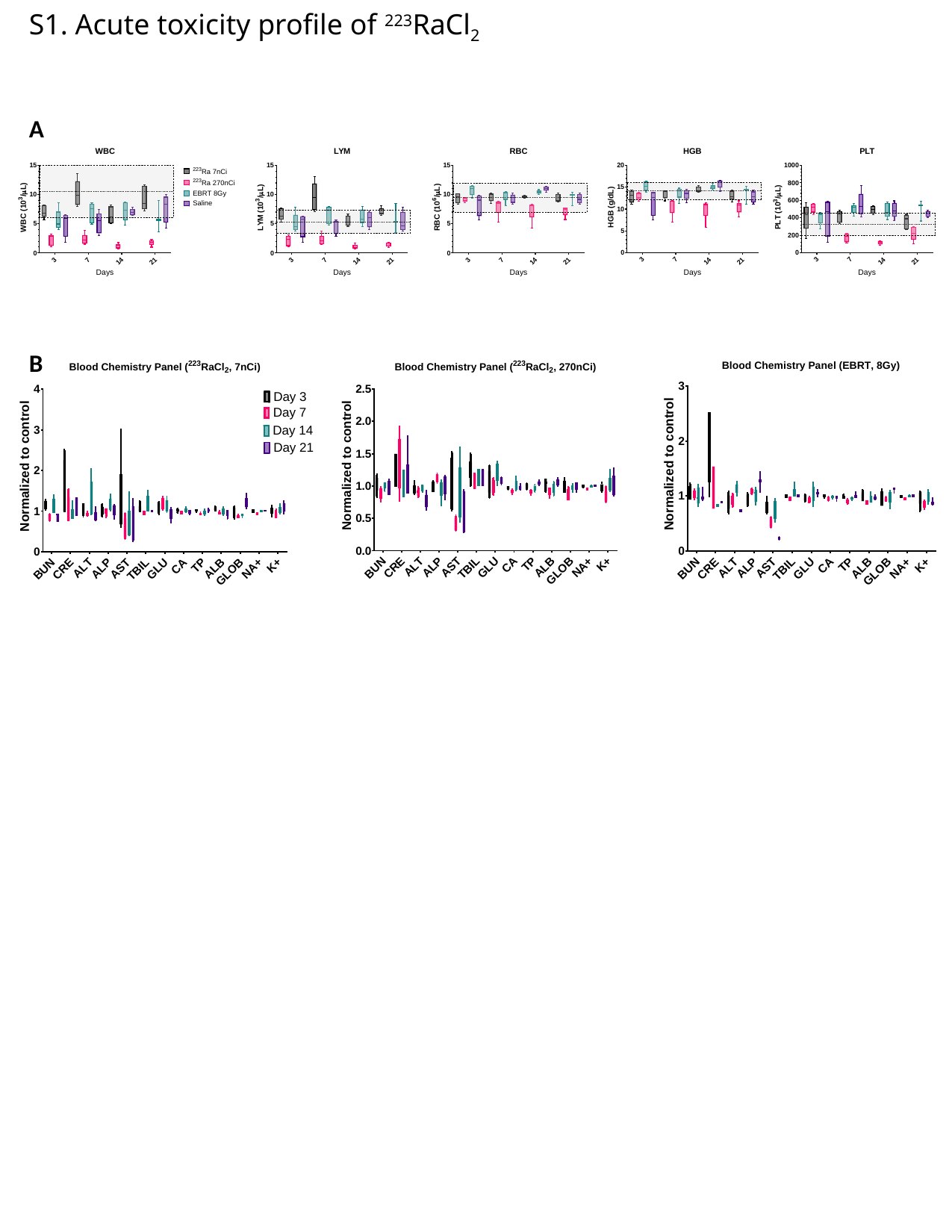

S1. Acute toxicity profile of 223RaCl2
A
B

## Slide 2
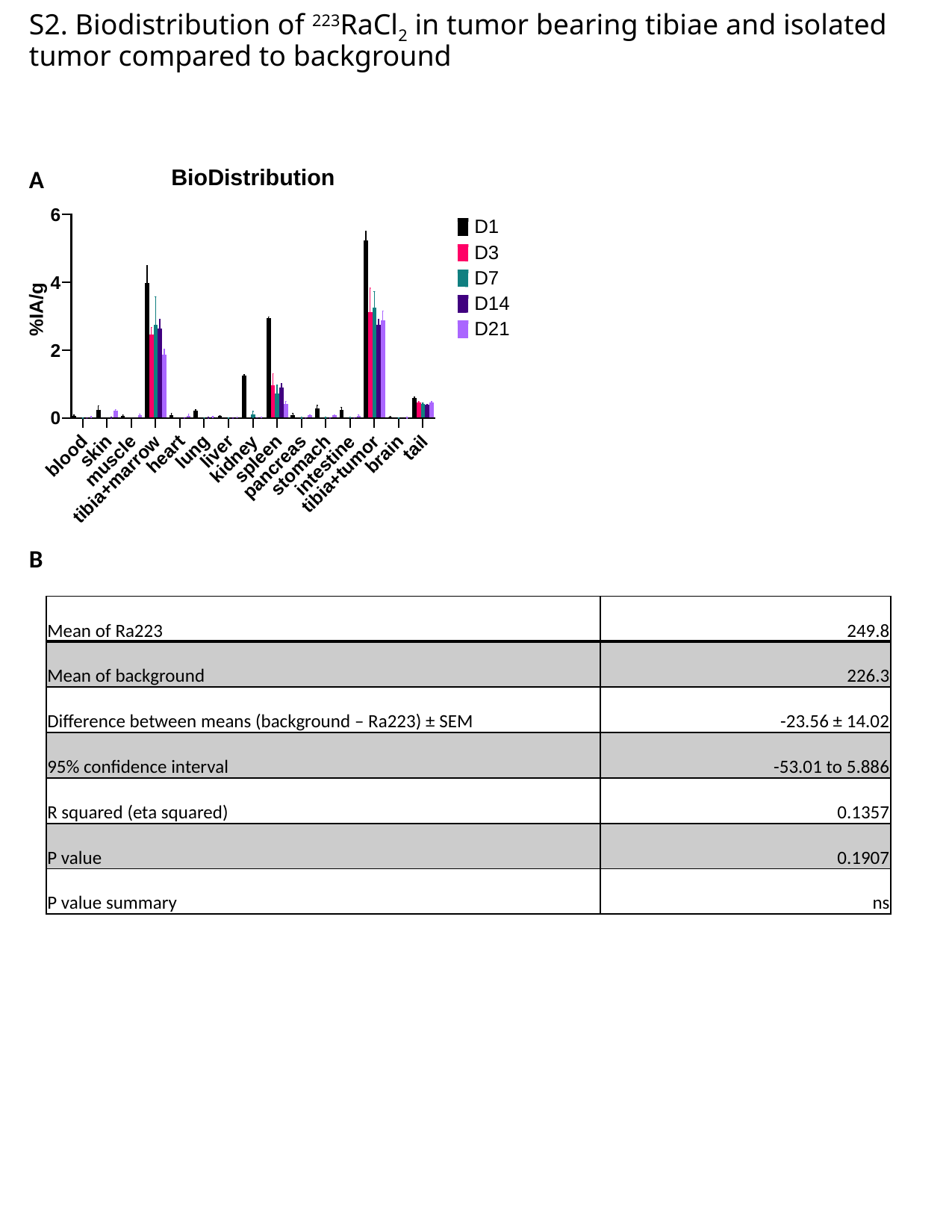

S2. Biodistribution of 223RaCl2 in tumor bearing tibiae and isolated tumor compared to background
A
B
| Mean of Ra223 | 249.8 |
| --- | --- |
| Mean of background | 226.3 |
| Difference between means (background – Ra223) ± SEM | -23.56 ± 14.02 |
| 95% confidence interval | -53.01 to 5.886 |
| R squared (eta squared) | 0.1357 |
| P value | 0.1907 |
| P value summary | ns |

## Slide 3
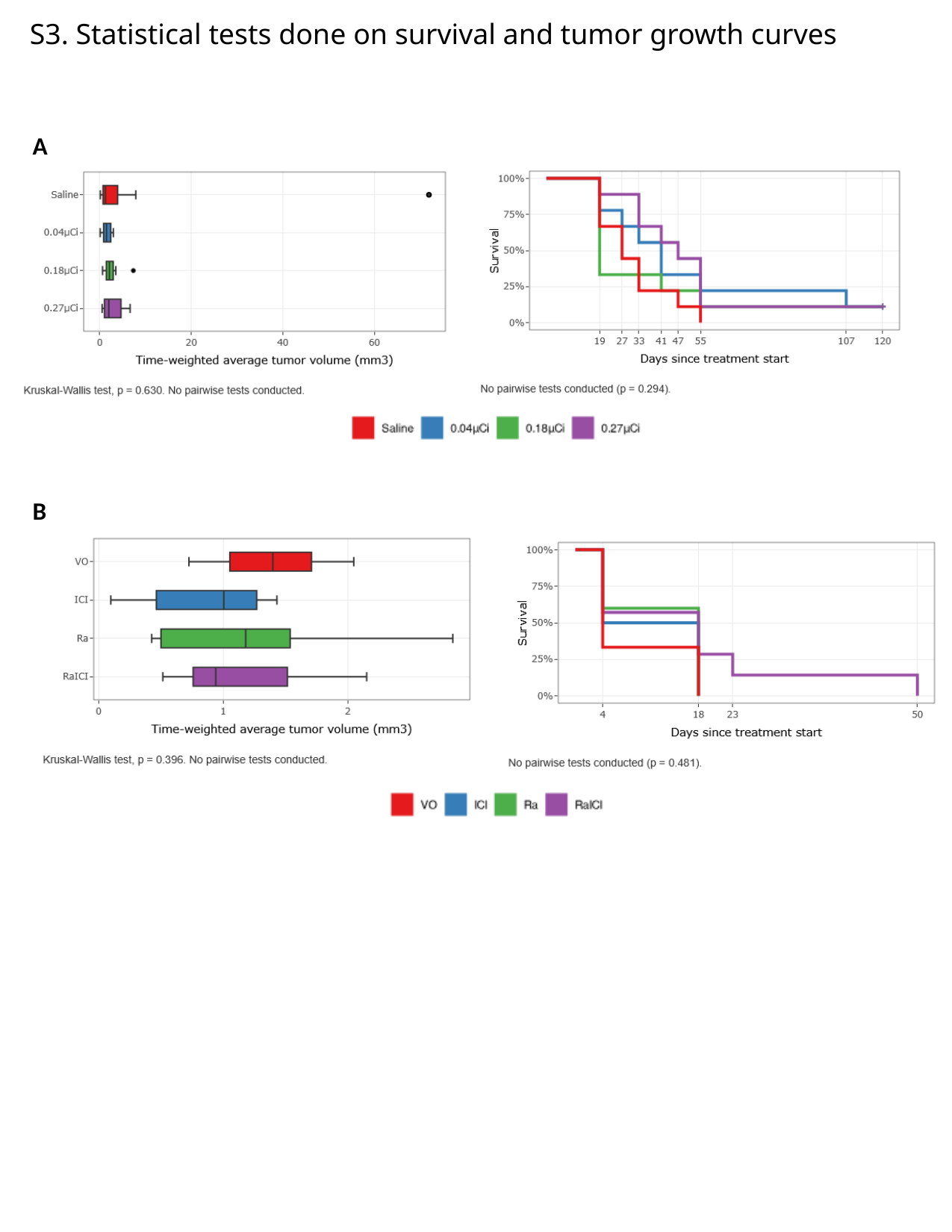

S3. Statistical tests done on survival and tumor growth curves
A
B

## Slide 4
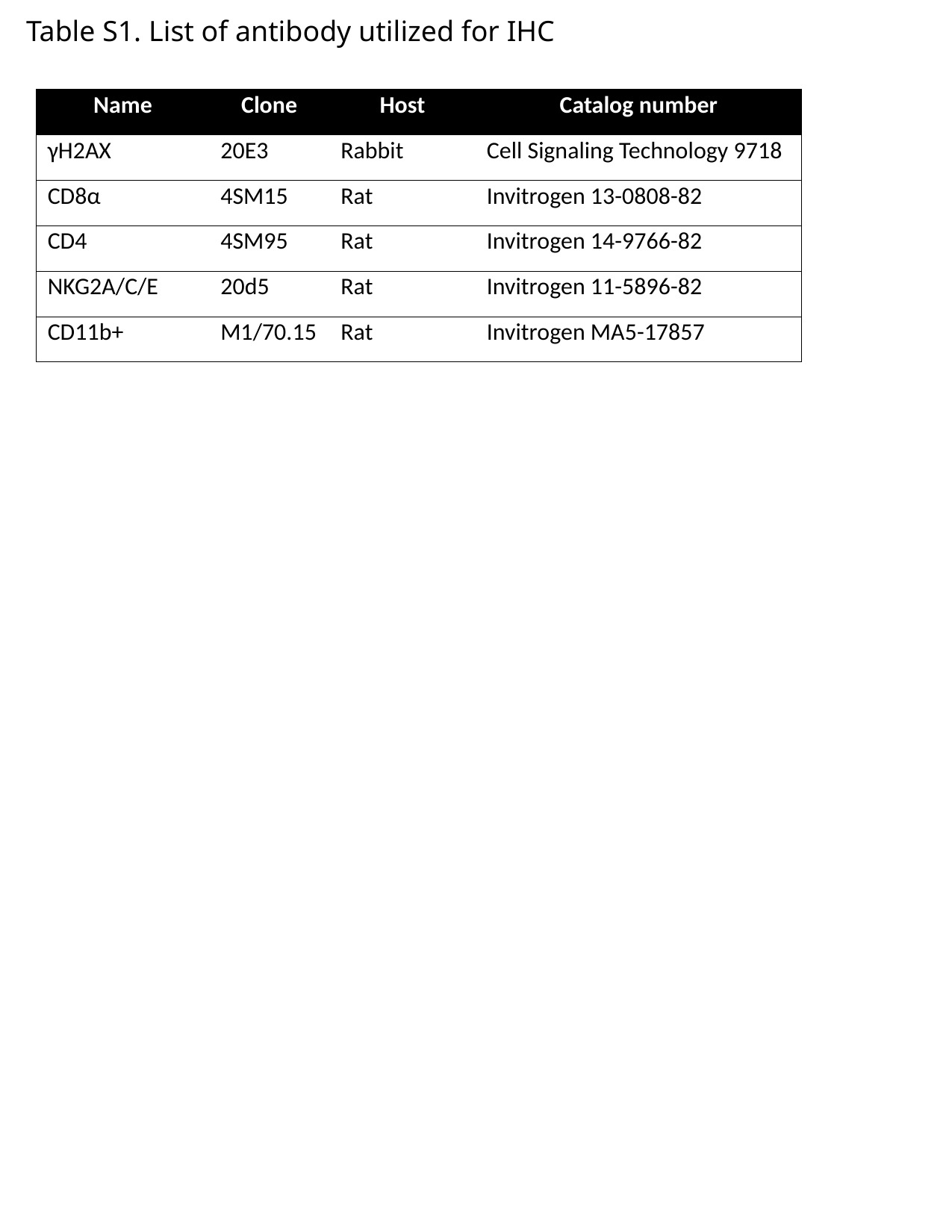

Table S1. List of antibody utilized for IHC
| Name | Clone | Host | Catalog number |
| --- | --- | --- | --- |
| γH2AX | 20E3 | Rabbit | Cell Signaling Technology 9718 |
| CD8α | 4SM15 | Rat | Invitrogen 13-0808-82 |
| CD4 | 4SM95 | Rat | Invitrogen 14-9766-82 |
| NKG2A/C/E | 20d5 | Rat | Invitrogen 11-5896-82 |
| CD11b+ | M1/70.15 | Rat | Invitrogen MA5-17857 |

## Slide 5
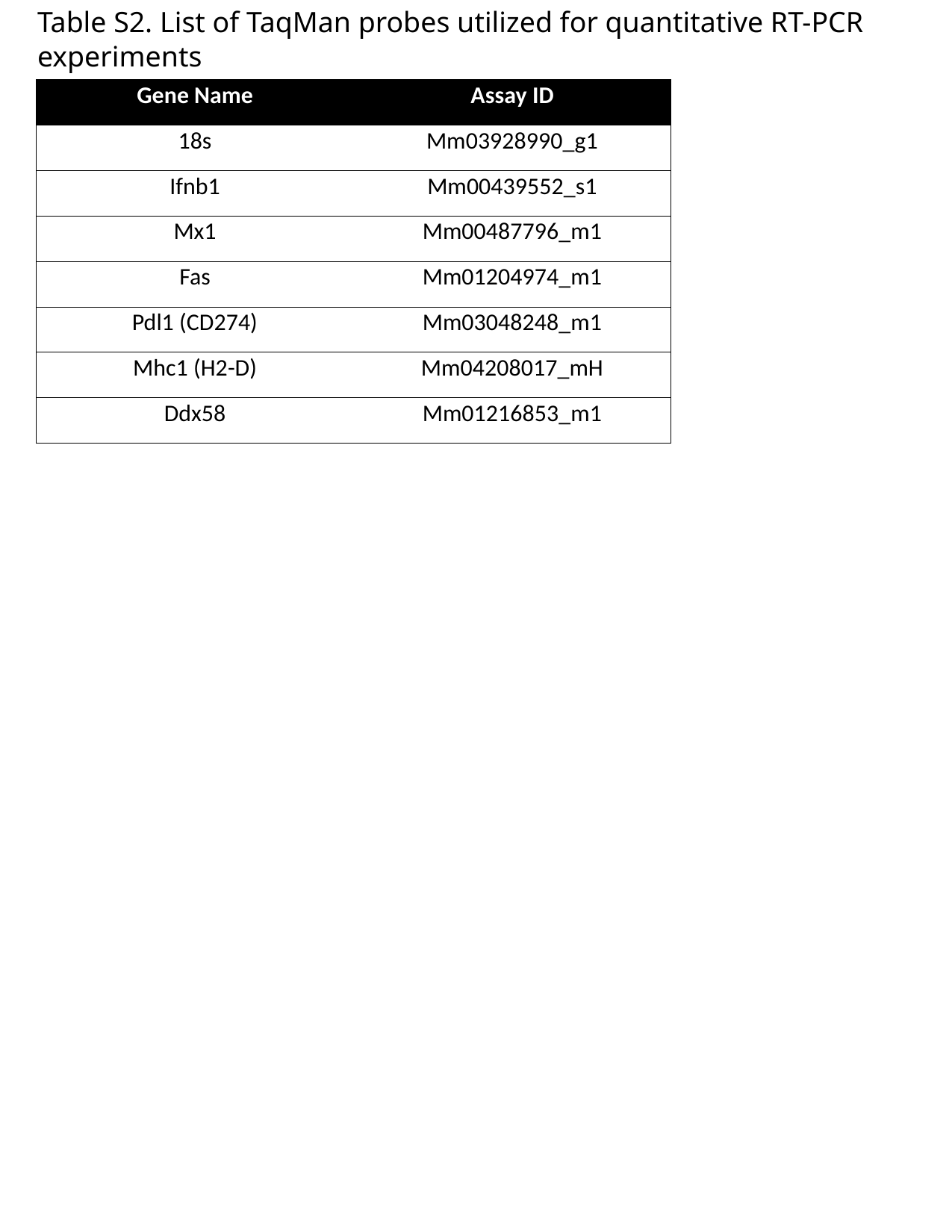

Table S2. List of TaqMan probes utilized for quantitative RT-PCR experiments
| Gene Name | Assay ID |
| --- | --- |
| 18s | Mm03928990\_g1 |
| Ifnb1 | Mm00439552\_s1 |
| Mx1 | Mm00487796\_m1 |
| Fas | Mm01204974\_m1 |
| Pdl1 (CD274) | Mm03048248\_m1 |
| Mhc1 (H2-D) | Mm04208017\_mH |
| Ddx58 | Mm01216853\_m1 |
